# Exploring the dynamics of a free fruit at work intervention

**DOI:** 10.1186/s12889-016-3500-4

**Published:** 2016-08-19

**Authors:** Amelia A. Lake, Sarah A. Smith, Charlotte E. Bryant, Sevil Alinia, Kirsten Brandt, Chris J. Seal, Inge Tetens

**Affiliations:** 1 Centre for Public Policy & Health, School of Medicine & Health, Wolfson Research Institute, Durham University, Queen’s Campus, Thornaby, Stockton-on-Tees, TS17 6BH UK; 2 Department of Nutrition, Technical University of Denmark, Mørkhøj Bygade 19, Søborg, DK-2860 Denmark; 3 Human Nutrition Research Centre, School of Agriculture, Food and Rural Development, Newcastle University, Newcastle upon Tyne, NE1 7RU UK; 4 Fuse—The UKCRC Centre for Translational Research in Public Health, Newcastle-upon-Tyne, UK

**Keywords:** Workplace, Fruit, Intervention development, Perceived healthy behaviour

## Abstract

**Background:**

The workplace has been identified as an ideal setting for health interventions. However, few UK-based workplace intervention studies have been published. Fewer still focus on the practicalities and implications when running an intervention within the workplace setting.

The objective of this paper was to qualitatively determine the perceived behaviour changes of participants in a free fruit at work intervention. Understanding the dynamics of a workplace intervention and establishing any limitations of conducting an intervention in a workplace setting were also explored.

**Methods:**

Twenty-three face-to-face interviews were conducted with individuals receiving free fruit at work for 18 weeks (74 % female). The worksite was the offices of a regional local government in the North East of England. Analysis was guided theoretically by Grounded Theory research and the data were subjected to content analysis. The transcripts were read repeatedly and cross-compared to develop a coding framework and derive dominant themes.

**Results:**

Topics explored included: the workplace food environment; the effect of the intervention on participants and on other related health behaviours; the effect of the intervention on others; participant’s fruit consumption; reasons for not taking part in the intervention; expectations and sustainability post-intervention; and how to make the workplace healthier. Five emergent themes included: the office relationship with food; desk based eating; males and peer support; guilt around consumption of unhealthy foods; and the type of workplace influencing the acceptability of future interventions.

**Conclusion:**

Exploring the perceptions of participants offered valued insights into the dynamics of a free fruit workplace intervention. Findings suggest that access and availability are both barriers and facilitators to encouraging healthy eating in the workplace.

## Background

The relationship between work, the workplace and individual health-related behaviours is an important area of research [1]. The workplace has been identified as an ideal setting for health interventions [2, 3] however few UK based workplace intervention studies have been published [4]. Fewer still focus on the barriers, practicalities and implications when running an intervention within the workplace setting [5, 6]. In England, the 2011 Public Health Responsibility Deal: Health at Work Pledges introduced by Public Health England (PHE) underpins the Department of Health’s core commitment to support the workforce to lead healthier lives [7]. One of the seven key priorities is the improvement of health in the workplace, and the health of those moving into and out of the workforce [8]. In June 2013 NICE launched a scoping consultation on ‘Workplace policy and management practices to improve the health of employees’ [9]. With rising rates of overweight and obesity, the workplace offers an ideal and unique setting to tackle diet and lifestyle behaviours which may modify energy balance [10–13].

Employment and the workplace environment are seen as significant contributors to food choice and eating behaviours [11, 14]. Workplace interventions have the potential to target a large proportion of the adult population and are an ideal setting in which to promote healthy lifestyles particularly as people spend a significant amount of their time at the workplace [3, 13]. There is evidence of a work–family spill-over and role overload which influences food choices [14] as well as evidence of a relationship between the perceived influence of employment and measured longitudinal dietary change [11].

A number of workplace-based interventions have attempted to change dietary behaviour [15–19]. Strategies such as education, counselling and alterations to the physical environment of the workplace have all been used in an attempt to modify dietary intake [20]. A number of systematic reviews into workplace interventions have shown that environmental modifications and education in relation to diet, physical activity and lifestyle factors have, in general, led to a moderate improvement in dietary intake [21–23]. The lack of evidence regarding the role of worksites and in particular the failure of many interventions to recognise and address the complexity of the work environment have also been acknowledged [22].

In the present workplace intervention the overall aim was to increase fruit intake by making it freely available and accessible to intervention participants, no other information was made available [24]. The focus on increasing fruit intake in this dietary intervention was because of the known health benefits of fruit consumption, particularly in terms of the inverse association between fruit intake and body weight [25]. The objective of this paper was to qualitatively determine the perceived behaviour changes of participants in a free fruit at work intervention. Understanding the dynamics of a workplace intervention and establishing any limitations of conducting an intervention in a workplace setting were also explored.

## Methods

Face-to-face interviews were conducted with participants in the intervention group of the Fruit at Work Study. The Fruit at Work Study was part of the ISAFRUIT project funded by the EU (http://cordis.europa.eu/pub/food/docs/biotech_isafruit.pdf); in this randomised controlled workplace intervention the overall aim was to increase fruit intake by making it freely available and accessible to intervention participants, in a simple, single factor intervention. The worksite selected was the offices of a regional local government in the North East of England, UK. This worksite was selected because of the large workforce, numbering over 1000 employees, all working very similar hours in an office environment, and housed within one building. Other factors included the building’s location on the periphery of a city with the majority of meals consumed on site. The region was selected as the North East of England has lower reported intakes of fruit and vegetables than more southerly regions [26]. In this intervention, 409 participants were randomly assigned, by building floor, to receive either daily access to free fruit (intervention group *n* = 206) or no fruit (control group *n* = 203) for 18 weeks (February 2009 - June 2009). The study recorded dietary intake (using a validated food frequency questionnaire (FFQ) [27]), anthropometric measurements of height, waist circumference, blood pressure, body weight and bio-impedance. This was done at baseline, at an interim point and at the end of the intervention for both the intervention and control group [28].

Throughout the 18 week intervention of free fruit provision, all intervention group participants who attended the ‘health checks’ during the interim measurements (week 9 of the 18 week intervention) were invited to take part. Email addresses were obtained from 46 individuals who were interested in taking part; 23 consented to take part in the interviews. The topic guide was developed following a review of the literature and discussions with the intervention team. All interviews were carried out in private during the working day. The interviews explored the food availability at the workplace, particularly in terms of its healthiness and the availability of fruit. Questions enquired about their participation in the intervention, exploring their work environment, and any perceived changes in behaviour as a result of the intervention, in particular if they were eating more fruit. The interviewer allowed the respondents freedom to expand on topics and interviews ranged in length from 20 to 35 min.

The study was conducted according to the guidelines laid down in the Declaration of Helsinki and all procedures involving human subjects were approved by the Newcastle University Research Ethics Committee (CL08/09/15). Written informed consent was obtained from all respondents.

### Data analysis

The interviews were recorded, transcribed verbatim and verified by the interviewer. The data were imported into the qualitative software package Nvivo 7 (QSR International Pty Ltd. Australia). The analysis was guided theoretically by Grounded Theory research [29] and the data were subjected to content analysis [30]. The transcripts were read independently by two researchers (AAL and CB) and cross-compared to develop a coding framework and derive dominant themes. Data saturation was reached at 23 interviews, with no new themes emerging, therefore no further interviews were conducted.

## Results

Twenty-three face-to-face interviews were conducted with individuals taking part in the intervention arm of the study; 17 were female and 6 male (73 % female compared with 60 % female in the intervention group). The interviews were analysed without relating them to any additional information about the interviewees, such as work role, educational background or socio-economic status. Nine key themes are discussed below.

### The workplace food environment and eating habits

Of those interviewed, many described bringing their own food into work with occasional visits to the workplace canteen or shop. Respondents indicated it was cheaper and more time efficient to bring their own lunch to work and avoiding queuing in the canteen. The canteen was associated with queuing, having cooked meals and lack of availability of healthier choices. Despite the perception that the canteen food had improved over time, there was an observation that the food was not of a good quality and was expensive. Fruit (not provided by the intervention) was available in the workplace but it was described as lacking in variety, expensive and not popular.

“Just not very much attention paid to what’s cooked I don’t think, and it’s just… I don’t think they’re fresh foods, very fresh.” [Female_4_I_FW218]

The lack of a microwave in offices and floor kitchens was seen as limiting food options at work.

The desk relationship with food emerged as a theme; described as a preferred and common venue for eating. Eating at desks limited the time taken on breaks so the workforce could take more time off or leave early using flexitime:

“…I think the issue is probably one of culture and how busy people are… you really need to eat at your desk because you’ve got so much work to do” [Male_2_I_FW153]

Another emergent theme was that of the office relationship with food. Many respondents discussed the wide availability of biscuits, cakes and sweets in offices despite the fact that many people were on weight reducing diets:

“… at birthdays people bring in some cakes or biscuits, if they’ve been on holiday they have to bring back some cakes or biscuits, and at Christmas time we all take turns of buying those big 1 kg tins. .” [Male_2_I_FW153]

Dieting was a common theme within the workplace, with colleagues following different weight loss regimes, and diets, food preferences and food a popular topic of conversation:

“we’re all quite focused at the moment, so we’re all sort of bringing salads in, eating our fruit, and yoghurts, you know, things like that, so I don’t know how we could become more healthy.” [Female_2_I_FW149]

### Perceived behaviour changes: Effect of the intervention on participants

The majority agreed that participation in the intervention meant they were eating more fruit. The start of the intervention was close to New Year, and for many respondents they had the ambition that it would herald weight loss. Additionally there was the perception that the fruit was helping them to start other healthy behaviours:

“Certainly from my point of view, I think, ‘Well, I’m doing this, I’m getting the fruit, let’s add it to,’ … ‘other lifestyle changes and let’s go out for a walk and let’s do more exercise and see what happens at the end of it.’” [Female_3_I_FW183].

Respondents acknowledged that they had not substituted fruit for other less healthy snacks:

“…but I’m also still eating the chocolate bars and still drinking the cans of pop. So I’m probably just eating more, not substituting.” [Male_2_I_FW153]

This was despite a perception that biscuits and chocolates in the office had decreased since the start of the intervention. Guilt was a reoccurring emergent theme associated with eating less healthy food and the need to eat more healthily:

“We feel a bit more guilty about eating chocolate and cakes all the time, or we eat fewer of them I think.” [Male_4_I_FW240]

However, for most, being part of the intervention appeared to be a driver for increased fruit consumption at work, with respondents feeling obliged to consume fruit at work, but not necessarily at home:

“I probably eat more fruit, definitely, because sometimes I would look at what I’ve got in the fruit bowl at home and think, ‘I don’t fancy any of those.’ But when it’s here and I feel like because I’m part of the study I think, ‘Oh, I should eat it, really,’ …” [Female_3_I_FW271]

A number of respondents commented that as a result of the intervention they were more likely to consume more fruit at the weekend ([Female_4_I_FW229] and [Male_4_I_FW422]).

“…at home I would never dream of just going to the fruit bowl and just picking an apple up, or picking a banana up, but now I’m actually starting to… if I’m having a cup of tea, after my cup of tea, rather than going for a biscuit I’m going to the fruit bowl.” [Female_4_I_FW217]

There was a common theme running through a number of responses that the fruit being provided in the workplace established a ‘habit’, which was easy to maintain outside of work:

“… I just seem to have got into the habit and to be honest I do enjoy it and there are even times when I’ve—especially when it’s come to holiday time and I’m thinking to myself, ‘I’ll have banana on Ryvita today instead of sandwiches,’ so probably eat more fruit and cut down on chocolate bars, because I certainly haven’t eaten as much of those.” [Female_4_I_FW215]

One female respondent described fruit as a weekday food which was not eaten either in the evenings or at the weekend [Female_2_I_FW150]. However, others did see the intervention as a driver to eat more fruit at home as well as at work, including for one, the purchase of a juicer to be used at home:

“… reducing the sweet things, and actually buying more fruit at home as well to kind of keep this up. “[Female_3_I_FW183]

Respondents suggested that taking part in the intervention had meant an increase in the variety of fruits they consumed.

Additionally, taking part in the intervention had highlighted fruit as an alternative snack option to chocolate bars.

Respondents discussed how the intervention had raised awareness around issues of healthy eating and health and how the intervention had a positive effect on them:

“… I’m filling myself up with fruit and I think subconsciously I’ve got that healthy frame of mind, ‘Don’t spoil it by eating a chocolate bar, have fruit today.’ So I think it definitely has had a positive impact. It’s how long I can keep it up for once we don’t get the fruit at work.” [Female_4_I_FW215]

As with the effect on others, guilt was described as a driver for the decreased consumption of other foods:

“Less biscuits and things, … Just guilt because there’s fruit there I think.” [Female_4_I_FW218]

The fruit, provided by the study, was perceived as providing a healthy option without which the workplace option would be “biscuits or nothing” [Female_4_I_FW218].

Respondents were reporting a range of benefits of free fruit, for example improvements in fitness (see section Other Health Behaviours). While respondents described the benefits of consuming the free fruit provided; the ‘medicals’, health checks’ or “weigh ins”, as the ‘measurement aspect of the intervention was frequently described and in particular being weighed was seen as an important aspect of the intervention.

Within offices, the health checks generated much discussion. Having their blood pressure and their anthropometric measurements recorded seemed to be a talking point:

“I think the medicals that we’ve had were comparing you know how’s your blood pressure been this time? Has it gone up or down? You know what’s your waist measurement? What’s your weight? Has that gone up or down? So I think amongst the group that’s taken part we’ve sort of shown a common interest in how we’re all doing and the effect of the fruit.” [Male_4_I_FW249]

Another emergent theme was that of peer support. In one male dominated office there appeared to be a lot of fun around the intervention. The office had fruit eating competitions and one office member described how they “ jilded [encouraged] each other on with the surveys” [Male_4_I_FW177]. The same respondent described how in their office a manager would encourage consumption through using sell-by-dates:

“… we’ve got an office manager who wanders around minesweeping the fruit he sort of cleans it up. He puts the sell by dates on the bananas last week. He writes biro sell by date and it’s still there a week later…” [Male_4_I_FW177]

### Perceived behaviour changes: Other health behaviours

In addition to eating more fruit respondents saw their participation in the study as a way to initiate other health behaviours Their consenting to take part in the study coincided with New Year resolutions:

“…So I said that I would cut the Coke out and try and cut down on the chocolate and biscuits and what have you, and try and eat a bit more healthily… and obviously the Fruit At Work kind of kick-started it.” [Female_4_I_FW217]

The opportunity to add other behaviours to the fruit intervention was discussed by a number of respondents:

“.. I think, ‘Well, I’m doing this, I’m getting the fruit, let’s add it to,’ …, ‘other lifestyle changes and let’s go out for a walk and let’s do more exercise and see what happens at the end of it.’” [Female_3_I_FW183]

One male respondent found the intervention made him more generally aware of what he was eating generally and he had started looking at food labels:

“… I find myself looking at the labels on stuff more just, you know, to think, ‘Well, how much is in that?’, or, ‘I wonder if they put salt in that,’ and I’ll find myself turning away stuff that normally I wouldn’t have thought twice about doing, and I don’t know it that’s just… well, it has to be something to do with it because I would never have really done that before.” [Male_4_I_FW422]

There were reflections that the intervention had not necessarily meant an increase in fruit purchases but for example, buying more low fat foods.

### Effect of the intervention on others

While interviews were not conducted with non-participants or the control group, increasing the fruit in the office was perceived to have influenced the behaviours of individuals not taking part:

“Somebody who’s in the office who sits opposite me always used to just buy a sandwich at the shop, now I’ve noticed he brings fruit in from work with his sandwich. Now I don’t know if that’s anything to do with it or not, but he does bring fruit in and he didn’t used to.” [Female_4_I_FW215]

Taking part in the study introduced some participants to new terms. For example, the concept of ‘body mass index (BMI)’, and male respondents described that they had started using scales in the office that pre-intervention only women had used. Another intervention office had a juicer—purchased by an office manager as there was surplus (uneaten) fruit in the office to juice.

The effect of the intervention on the office culture was described as making the office feel “more guilty” in terms of consuming cakes and sweets [Male_4_I_FW240]:

“…, at one time we were sort of having snacks, the cakes and the sweets, on a regular basis, but I think we feel more guilty about doing it now, and obviously if you’ve got fruit to eat anyway it takes the edge off your hunger pangs …” [Male_4_I_FW240]

When asked to reflect on the effect of the intervention it was perceived that it may *not* have influenced their dietary behaviour, but that it had made them think about their food intake:

“… it’s made everyone think a little bit more about what they eat, because you’re having to record it and that kind of thing… so all it’s done for me is meant that instead of buying fruit and bringing it in, I’ve just had it handed out, which is great, free of charge.” [Male_2_I_FW357]

Aside from their colleagues, respondents noted that their partners and children were eating more fruit as a result of them taking part in the study. For some this was because they were taking extra fruit home, but for others it was about eating fruit at home in front of children and setting a good example.

### Fruit consumption

From this sample of 23 intervention participants, only two reported that they did not consume fruit prior to the intervention. There was a large proportion of the respondents who perceived the study as an opportunity to have the fruit that they were already consuming supplied to them without incurring any cost. In many ways the intervention was selective; respondents indicated that only those interested in eating fruit or who liked fruit were taking part and taking advantage of free fruit provision:

“I think those I’d never seen eating fruit at all didn’t tend to show much interest in the study but that might just be the fact that they don’t like fruit. Those that occasionally brought fruit in are probably eating a bit more now than they used to. And those that used to bring a lot of fruit in tend to bring the same amount in to be honest, or less because it’s going to be supplemented by the study.” [Male_4_I_FW249]

“I don’t know if it’s changed practice because I would generally eat a couple of pieces anyway, so all it’s done for me is meant that instead of buying fruit and bringing it in, I’ve just had it handed out, which is great, free of charge.” [Male_2_I_FW357]

However, others acknowledged that the fact that the fruit was free, it encouraged increased consumption:

“‘This is free, great, I’ll eat more of it.’” [Male_4_I_FW172]

### Reasons for not taking part

Intervention participants were asked why they thought colleagues had not taken part in the intervention. There was a range of reasons given including reflections on personal characteristics. For example in an office where the majority had taken part there were two who did not:

“There’s **, and he says he doesn’t volunteer for anything, and ** who is 61, who says he can’t be bothered, and he’s overweight. [Male_4_I_FW174]

“… the ones who don’t do—well, as far as I know, don’t do a lot of physical activity, haven’t taken part in it” [Male_3_I_FW404]

In another office there appeared to be a range of reasons for not taking part from missing the registration through to only liking prepared fruit:

“Some just didn’t sign up in time, or missed the boat. We talked about it, but hadn’t got round to registering. Others don’t particularly like fruit, so they didn’t want to do it. And there was one girl who does like fruit, but she likes it all to be prepared for her.” [Female_3_I_FW183]

### Expectations after the intervention ends

There were some realistic perspectives about reverting back to normal behaviour post-intervention and maintaining motivation:

“Probably revert back to biscuits actually. ” [Male_4_I_FW177]

“It might tail off, I don’t know. … The intention’s there, but it’s whether or not the motivation lasts, probably.” [Female_4_I_FW215]

Trying to maintain the healthy behaviours developed during the intervention seemed important to some participants:

“I intend to try and snack on the fruit and the yoghurts and things at work, as opposed to the biscuits and the chocolate… I won’t suddenly go back to drinking the Coke—that’s something in my mind that says, ‘That’s a treat’ now.” [Female_4_I_FW217]

### Sustainability of the intervention

Most respondents were aware that their employers were not paying for the fruit and agreed it would be a very good idea to continue with the free fruit at work. However, they perceived it would be unrealistic. An emergent theme of the fact that their employer was publically funded was dominant in the discussion around the sustainability of the intervention:

“… we’re paid for through public funds and why should taxpayers pay for us to have fruits, I don’t think they should, at the end of the day we’re in jobs, we can afford to buy fruit ourselves. There are a lot of people out there at the moment not in jobs and they could probably do with the fruit more than what we could.” [Male_2_I_FW153]

Given that the expectation to receive free fruit would not be realistic, one respondent suggested that the council provide more fruit in the canteen and at a reduced price.

### How to make the workplace healthier

Respondents gave suggestions of how to improve health and eating behaviours at work. There was an acceptance that with flexi-time, people were not going to want to ‘waste’ time getting food. Therefore the solution would be to make desk-based eating healthier, which emerged as a theme. The key to successful desk based eating required minimum preparation:

“You can definitely have healthy food at your desk. But I think the key is it’s got to be pre-prepared. You don’t want to have to try and find a knife and fork or a sharp knife to cut something and prepare the food at your desk” [Male_2_I_FW153]

Another suggestion was the replacement of the tea-trolley with a fruit trolley, where workers are a ‘captive’ audience and the trolley provides healthy options and brings fruit to all floors of the building. One respondent suggested that having someone monitor their weight and BMI made them more conscious of their behaviours:

“What’s to stop trolleys coming round with fruit if they want to keep people healthy at work? [Male_4_I_FW174]

“I’ve never really taken any notice of BMI or what my body fat or waist size, apart from buying clothes. So it’s kind of interesting getting that over a period of time, and it does make you think, ‘Oh, yeah, I want to try and keep that down and make sure it doesn’t increase.’” [Male_2_I_FW357]

Suggestions to improve the healthiness of the workplace included making healthier options available at the canteen and to have a greater variety of fruit available.

“…I definitely think rather than just the bog-standard fruit bowl, you know, with your banana at 50p and your apple at… maybe if there’s a bit more variety in the fruit?” [Female_4_I_FW217]

“It’s just your apples, your oranges, the odd pear and bananas… there is always that… when it’s plonked in a fruit bowl it doesn’t inspire you to eat it…” [Female_4_I_FW217]

Additionally to subsidise the healthier options within the workplace canteen.

There were a number of comments around increasing the availability and variety of foods available in the canteen:

“…the main thing would be if the canteen could offer us more proper fresh vegetables rather than vegetables, you know canteen vegetables that, over necessity, have to sit there for longer than you would like them to.” [Female_4_I_FW261]

There were suggestions that biscuits needed to be removed and the shop that sold scones needed to be taken away [Female_4_I_FW229], while another suggested a “ban” on chocolate and cakes [Female_2_I_FW436]. A number of respondents suggested the workplace continue to provide them with free fruit, however all felt this was unrealistic (see section ‘Sustainability’)

Respondents suggested that workplaces encourage peer support and teams of people in place who could be physically active together:

“…I just think it helps with motivation if you’ve got a group of people doing stuff. So that office environment can help motivate you, it’s that kind of group thing, isn’t it?” [Male_2_I_FW357]

While respondents acknowledged they got preferential membership at a nearby gym, there were suggestions that free gym membership or free classes would be a good motivator.

## Discussion

This paper has described the perceptions of a sample of participants towards the end of an intervention where free fruit was distributed in the workplace. Findings highlight that access and availability are both barriers and facilitators to encouraging healthy eating in the workplace; barriers being lack of access to cooking facilities, the perceived availability of unhealthy options in the canteen or shop, and the high availability and ease of access to cakes and confectionary in the office. Facilitators included increased access and availability to fruit in the workplace, which is in line with existing evidence [24, 31] . Suggestions to increase workplace health included improvement of the canteen food and cooking facilities, mandatory health checks and the continued provision of free fruit.

### Perceived behaviour changes

Perceived behaviour changes included trying the new foods that the study had introduced to them. This included being more adventurous with the foods they purchased, not only for them but also for their family. The interviews suggest that by increasing the availability and making fruit more accessible to the intervention group, participants were eating more fruit and non-participants were also eating fruit with increased regularity, compared with before the intervention. The intervention made fruit highly visible within the workplace which was perceived with positivity. There were descriptions of ‘guilt’ associated with consuming less healthy foods, which needed to be balanced with healthier eating and behaviours.

While many respondents perceived they were eating more fruit as result of the intervention, few were strongly convinced they could continue with this behaviour once the intervention stopped. There were comments that the usual office food environment and the office relationship with food was a case of ‘biscuit or nothing…’ and that if an individual had forgotten their fruit they were unlikely to go to the canteen for fruit (despite all largely acknowledging that the canteen did have fruit available). Some felt they would continue with increased portions of fruit but the practical challenge of availability remained.

The interviews illustrated that the majority were not substituting energy dense snacks, such as crisps and chocolate, for the fruit provided by the study; which contrasts with the aims of the intervention and existing evidence [24] and merits further exploration in the quantitative study [28]. It also suggests that the simple intervention of improving access and availability of fruit may not be enough, and individuals may need further information and interventions.

### The dynamics of a workplace intervention

One major difference between a workplace intervention and an individual intervention is the potential for peer effect [32]. Colleagues within a shared workspace spend a lot of time in each other’s company and show an intricate knowledge of each other’s behaviours and motivators for behaviours. The interviews revealed that behind the scenes in the offices there were a range of peer-effects and interactions between colleagues due to the fruit study. Managers within the offices also had a role, including managers who purchased juicers for the office and those who wrote sell-by-dates on fruit left sitting around. These managers appeared to have an important role in encouraging teams to take part in the intervention. In future interventions it would be useful to try to capture this peer effect to determine its influence on the success of an intervention [32]. There is some evidence of the importance of engaging worksite teams in the implementation phase [33].

Time was an important factor in relation to workplace food choices for these participants and time is acknowledged as an important factor in influencing food choice [11, 34–36]. One benefit of the intervention was that fruit was delivered to their floor and no time was ‘wasted’ getting fruit. The results presented here agree with a review by Jabs and Devine [35] where choice of food was influenced by how much time was available. Desk-based eating was popular in order to leave work earlier (to have more family time) or to do errands. There is a lack of evidence around time issues, food choices and work productivity as well as employee satisfaction which merits further investigation [35].

Most respondents appeared to bring their own lunch food into work. In terms of food availability and accessibility, the workplace canteen perceived negatively and many respondents would have preferred better ‘in-office’ facilities (such as a microwave) for heating up their own food. When suggesting ideas to improve the healthiness of their workplace many individuals focused on having healthier choices and a wider range of choices available at the canteen, for example a greater variety of fruit at a subsidised cost. Other studies have also found a lack of healthier options in the workplace and limited options of more traditional foods [37].

Participants perceived that those who consented to take part in the study were already consuming fruit and had an awareness of healthy eating. Out of a sample of 23 intervention participants, only two were interviewed who *did not* consume fruit prior to the intervention. Many described substituting the fruit they would have already brought into work with the fruit provided by the study. Only a minority described eating the two portions of fruit in addition to their current fruit intake (i.e. having 3–4 portions of fruit per day). The interviews suggested that intervention participants were taking fruit home with them when they could not eat it at work, and this may indicate that other family members were eating more fruit, which may have been the fruit provided by the study. The study did not measure the fruit intake of other family members, using food frequency questionnaires measured the fruit intake of the participants [28].

The ‘weigh-ins’ or ‘health checks’ were seen as an important aspect of the intervention and provided a conversation point in the office, as has been discussed in the results. They seemed to be more of a talking point than the provision of the free fruit to the workers. An emergent theme of ‘males and peer support’ illustrated that male dominant offices showed friendly competition regarding weigh-ins. Additionally the intervention increased male awareness of terms such as BMI whilst changing their behaviour in terms of weighing, ‘that it’s not just for the women’.

### Limitations of conducting an intervention in a workplace setting

The comments offered by the respondents provides a much needed platform from which to design future workplace interventions, including the suitability of the type of intervention e.g. that in the political climate at the time of the present study, free fruit provision was not acceptable by a publically funded body. One of the key lessons learnt from this workplace intervention, gained not only from the interviews, but also from other sources of information during the intervention process, was the importance of having the support of key individuals in the workplace. The importance of the formative phase of the research in the workplace has been emphasised [10, 33]. While many studies are US based, there are many lessons which can be applied to other countries [10]. For example, during the formative phase of the present study, only the head building manager was included in the discussions, and not the two managers, nor the Head Porter or their team who actually booked rooms and controlled the building. This oversight then developed into difficulties, for example when rooms had to be booked for the health checks with 400 individuals. On reflection, we believe this is because at an early stage they were ordered to accommodate us from a higher authority and were not involved from the beginning in the design of the study or the logistics of the study design. However, despite their late involvement in the study design and implementation, once the intervention started the Head Porter and his team of Porters became ‘champions’ and the intervention would not have been possible without their aid and assistance.

### Limitations of this study

While a relatively small number of interviews (*n* = 23) were conducted with individuals from the intervention group (*n* = 206) who attended the ‘health checks’ during the interim measurements, our analysis indicated that data saturation had been reached. However if it had been possible, we would ideally have conducted further interviews with the intervention participants following the intervention and also with individuals from the control group to try and explain differences between the two groups. The majority of participants in the intervention arm of the study were female, similar to the intervention. A higher number of males could have highlighted a difference in perceptions. As with the reporting of food intake, discussion around fruit intake may have been subject to social desirability and social approval biases. Respondents acknowledged that only people already eating fruit were taking part in the wider study.

## Conclusions

This research has highlighted participants’ perspectives on an intervention in the UK workplace setting, adding considerably to the few qualitative studies that exist around food in the workplace. Using qualitative methods to complement a largely quantitative study adds breadth and depth to the findings. It offers insight into the dynamics and complex interactions occurring behind the scenes, which have the potential to limit the success of an intervention in a workplace. Individuals had perceived some behaviour changes related to their participation in a fruit intervention, changes included increases in the variety of fruit consumed, healthier eating, increasing physical activity. Some reported eating more fruit at home, while a minority saw fruit as a week-day food. Participants had concerns about the sustainability of these changes once the intervention stopped. The potential for peer influence within a workplace intervention (around fruit consumption and health checks), the importance of time and practical aspects such as liaising with the Porters are important factors to consider when designing a workplace intervention. These findings will be used to help design and develop future workplace dietary interventions.
